# Study of the Reliability and Validity of Objective Structured Clinical Examination (OSCE) in the Assessment of Clinical Skills of Audiology Students

**DOI:** 10.5539/gjhs.v5n3p64

**Published:** 2013-01-31

**Authors:** Mansoureh Nickbakht, Marzieh Amiri, Seyed Mahmoud Latifi

**Affiliations:** 1 Rehabilitation Administration, Musculoskeletal Rehabilitation Research Center, School of Rehabilitation Sciences, Ahvaz Jundishapur University of Medical Sciences, Ahvaz, Iran; 2 Department of Audiology, School of Rehabilitation Sciences, Musculoskeletal Rehabilitation Research Center, Ahvaz Jundishapur University of Medical Sciences, Ahvaz, Iran; 3 Department of Statistic and Epidemiology, School of Health, Diabetic Research Center, Ahvaz Jundishapur University of Medical Sciences, Ahvaz, Iran

**Keywords:** educational measurement, students, objective structured clinical examination, reliability

## Abstract

**Introduction:**

Audiology students should possess clinical competence and skills. To achieve this, their clinical skills must be properly assessed. The Objective Structured Clinical Examination (OSCE) is a standard and fair examination of clinical competence. The goal of this study is to devise a checklist of OSCE examination criteria and study their validity and reliability for assessing the clinical competence of Audiology students.

**Methods:**

Among the various procedures in which audiology students should possess demonstrated competence, 10 specific skills were selected and checklists were prepared. Faculty members of university’s Audiology Department were consulted to determine the validity of the checklists. Subsequently, the examination was administered to all 14 fourth-year audiology students in their final semester of study at Ahvaz Jundishapur University of Medical Sciences. The examination consisted of three question stations and seven procedure stations. Each station was managed by two examiners who independently used a checklist to score each student’s performance in a given procedure. To determine reliability, the Spearman test was used.

**Results:**

The correlation between each examiner’s scores of students at question stations was 0.908. The correlation between each examiner’s scores at procedure stations was 0.857 (p=0). The site of lesion test had the highest correlation (0.948) and immittance audiometry had the lowest correlation (0.585).

**Conclusion:**

The prepared checklists had good validity and reliability and can be used to evaluate the clinical competence of audiology students in their final semester of study.

## 1. Introduction

Today, new methods, such as the Objective Structured Clinical Examination (OSCE) are used instead of traditional pen-and-paper tests to assess students of medical sciences (Turner & Dankoski, 2008). Traditional written or oral exams can only assess clinical knowledge while the OSCE tests both knowledge and skill (Bhatnagar, Saoji, & Banerjee, 2011). This examination, which is structured in different “stations”, assesses a student’s clinical and technical skills. In this exam, decision-making strategies, problem-solving abilities, and critical thinking are measured (Silva, Lunardi, Mendes, Souza, & Carvalho, 2011). In the OSCE, the student must perform specific activities in a fully structured clinical environment within a specified time (Zraick, Allen, & Johnson, 2003; Samira & Amer, 2012). Miller proposes a model for measuring each examination. Based on this model, four levels are assessed:

- Knows
- Knows how
- Shows how
- Does

Traditional written exams can only assess the first two levels. The fourth level is assessed only in an actual clinical setting with a real patient. However, the OSCE method can assess a student’s skill at the third level (*i.e*. demonstration of the student’s ability under objective and standard conditions) (Rushforth, 2007). In this examination, Satisfaction of students is higher than traditional or practical exams (Bolhari, 2007) and students spend more time studying for the OSCE (Mavis, 2000). The most important disadvantage of this method is the higher level of stress that some students can experience (Rushforth, 2007). In each of the OSCE stations, a clinical skill is tested using an appropriate checklist. Therefore, it is necessary to assess those checklists in terms of their reliability and validity. In a review paper, after underscoring the importance of the OSCE method, Chumley has emphasized that checklist validity and reliability must be studied and confirmed before the administration of the OSCE (Chumley, 2008).

Bachelor’s students of audiology in Iran refer to hospital audiology clinics and rehabilitation centers from semesters three through eight and complete internships under the supervision of trainers. During these periods, students are trained to perform audiology tests, interpret findings, consult patients, and apply in practice on patients what they have learned. At the end of the fourth year, the student is expected to be able to perform audiology evaluations. In addition, the student is expected to correctly interpret audiometry results and give necessary and appropriate consultations to patients. Previously, the final student evaluation was performed by faculty members through oral questions. However, since 2012, the OSCE method has been used. In this method, the student is evaluated when in different realistic clinical settings. To take examination through the OSCE method, appropriate checklists must be prepared and evaluated. The aim of this research is to study validity and reliability of OSCE checklists and methodsfor evaluating clinical skills of the students.

## 2. Materials and Methods

In this cross-sectional study, the examination was designed in 10 stations for fourth-year audiology students at Ahvaz Jundishapur University of Medical Sciences. First, a list of all audiology clinical skills that a graduating student must learn in his internship needed to be prepared. For this study, 15 clinical skills were identified. Then, the lists were provided to faculty members of the Audiology Department of Ahvaz Jundishapur University, who were asked to prioritize the skills based on importance and applicability. After collecting their perspectives, the researcher selected the10 highest-ranked procedures: “Case history, Tuning fork tests, Otoscopy exam, Audiologic interpretation, Differential diagnosis, Immittance audiometry, Puretone audiometry, Clinical masking, Site-of-lesion tests, Decision-making, and Consultation. Next, checklists of each procedure were designed by studying the OSCE examinations, and academic texts. To determine the face validity of these checklists, we asked faculty members of the Audiology Department (6 person) to review the checklists in a debrief meetings to discuss their perceptions. Considering the experienced viewpoints and perspectives of them, revised checklists were prepared. In this regard, 65 skills were included in 10 checklists. Two types of stations were designed for this study:

1) Seven procedure stations
2) Three question stations

All 6 faculty members agreed with final checklist items which indicated that checklists had good face validity.

Three employees and a member of the rehabilitation faculty were used as standard patients and were trained before the day of the examination. All faculty members and clinical educators of audiology were selected as examiners and two examiners were present in each station.

For procedure stations, two examiners observed the performance of the student, but question stations were controlled by just one observer. (However, the student’s answer sheet from the question stations was evaluated by two examiners). A few days before the examination, a session was held for all 14 fourth-year audiology students. The procedure and its goals were explained and information regarding the attendance and general format of examination was provided to the students. One day before examination, all examiners received their evaluation checklists and related remarks in a session. Each examiner recorded his evaluation of the student’s performance on a separate score sheet to measure the reliability of the checklists.

The examination was given to all 14 fourth-year audiology students. The time at each station was five minutes. Each student performed the requested activity after arriving at the station and studying the relevant instructions. At each station, the examiner observed and timed the student’s performance and scored each item from the checklist with a zero or one. Then, the student was referred to the next station.

At the end of the examination, the checklists were collected and data was entered into SPSS software. Regarding the reliability of an examination, one important point is the accuracy of that examination. To determine accuracy, term inter rate reliability (IRR) was used. To determine IRR in the OSCE method, each of the two examiners’ scores at each station were compared. In most studies, correlation is used for measurement. In correlation, zero means the absence of a relationship and one means full agreement between two studied phenomena. To determine reliability of this examination, the reported scores of both examiners were studied at each station. The correlation of total scores reported by the two examiners was studied. The Spearman test was used to check the inter rater reliability in the analysis.

## 3. Results

The face validity of the checklists was confirmed by audiology faculty members in a meeting. They suggested that checklists need a little too details. Their point of view was done in checklists. Finally all of the 65 items in the checklists meet 100% agreement and the face validity was good.

To determine the reliability of the checklists, the correlation between reported scores of the two examiners was separately calculated for each of the 10 stations, which ranged from 0.58 to 0.94. The correlation between total student scores was determined by the first and second examiners (p=0.0) (see Table 1). Correlation between scores of student was calculated from the question stations (stations 4, 5, and 10) from the first and second examiners (r=0.908 and p=0.0). The correlation of student scores was calculated from procedure stations relating translations 8, 7, 6, 3, 2, 1 and 9 (r=0.857, p=0.0).

**Table 1:** Correlation between examiners scores

Station Number	Station title	Correlation	P-value
**1**	case history	0.743	0.002
**2**	tuning fork tests	0.829	0.0001
**3**	otoscopy	0.893	0.0001
**4**	audiologic interpretation	0.791	0.0001
**5**	differential diagnosis	0.922	0.0001
**6**	immittance audiometry	0.585	0.028
**7**	puretone audiometry	0.936	0.0001
**8**	clinical masking	0.854	0.0001
**9**	site-of-lesion tests	0.948	0.0001
**10**	decision-making and consultation	0.794	0.001
**Total**		0.846	0.0001

## 4. Discussions

As a general evaluation of efficacy of the designed stations, it can be said thatthere is a good, significant correlation between views of examiners (r=0.84 and p=0.0). Some studies report a correlation of 0.6 and above as a good correlation and consider correlation 0.8 as a good standard (Rushforth, 2007).

In a study of 175 first- and third-year nursing students, correlation of the OSCE method was reported at 0.53-0.99, which had the lowest correlation coefficient of 0.5, similar to this study (Rushforth, 2007). Correlation between the examiners’ assessments at question stations was higher than correlations between examiners at procedure stations. Perhaps this is due to having enough time for evaluating answers at question stations by examiners, which can cause accurate evaluation, because the question stations were evaluated after the examination within sufficient time.

In another study, Moattari, et al. (2007), included both examiners in each of 10 stations to study the reliability of the OSCE method and evaluated students across two consecutive days. The correlation coefficient of scores given by two examiners was desirable (r=0.96 and p<0.001). Results of that study were better than results of the correlation obtained from our research (r=0.84 and p=0.0). Of course, Moattari’s study reported a very low correlation coefficient (0.38) for one examination station (wearing surgical gloves).

In our study, only immittance audiometry station (r=0.585 and p=0.028) failed to score a high correlation, indicating that questions for this station was not easy to differentiate and they should be reviewed.

Perhaps low correlation at this station is due to the small sample size and the number of students that was only 20 in some studies, such as those conducted by Hussein et al. and Jain et al., but the correlation was higher than 0.7 (Rushforth, 2007). For the immittance audiometry station, students should have performed tympanometry and acoustic reflex test in one ear of the standard patient. In this station, students should have provided explanations to the patient and recorded the result after performing the examination. The checklist for this examination station contained eight items. Of course, low correlation may also be due to the time limitation of this station because Gupta believes that the brief time that is necessary for a station can lower reliability of the examination (Gupta, Dewan, & Singh, 2010).

In This study the faculty member feedback suggested that the checklists had good face validity. Similar to our study, Macluskey et al. (2011) examined face validity of OSCE checklists in a meeting (with members of the education committee of the Association of British Academic Oral and Maxillofacial Surgeons) and their checklists had validity too.

## 5. Conclusion

Results of this study showed that nine items out of the ten audiology OSCE checklists had good reliability but immittance checklist need revise. If more stations, longer times, or more students are used in subsequent studies, one can better evaluate the ability of students and the reliability of the checklists.
